# Modification of Chitin with Kraft Lignin and Development of New Biosorbents for Removal of Cadmium(II) and Nickel(II) Ions

**DOI:** 10.3390/md12042245

**Published:** 2014-04-10

**Authors:** Marcin Wysokowski, Łukasz Klapiszewski, Dariusz Moszyński, Przemysław Bartczak, Tomasz Szatkowski, Izabela Majchrzak, Katarzyna Siwińska-Stefańska, Vasilii V. Bazhenov, Teofil Jesionowski

**Affiliations:** 1 Institute of Chemical Technology and Engineering, Faculty of Chemical Technology, Poznan University of Technology, M. Skłodowskiej-Curie 2, Poznan 60965, Poland; E-Mails: wysokowski@wp.pl (M.W.); lukaszklapiszewski@tlen.pl (Ł.K.); przemyslaw.bartczak88@gmail.com (P.B.); tomasz.szatkowski88@gmail.com (T.S.); majchrzak.iza@gmail.com (I.M.); katarzyna.siwinska-stefanska@put.poznan.pl (K.S.-S.); 2 Institute of Inorganic Chemical Technology and Environmental Engineering, West Pomeranian University of Technology, Pułaskiego 10, Szczecin 70322, Poland; E-Mail: dmoszynski@zut.edu.pl; 3 Institute of Experimental Physics, TU Bergakademie Freiberg, Leipziger Str. 23, Freiberg 09599, Germany; E-Mail: vasilii.bazhenov@physik.tu-freiberg.de

**Keywords:** chitin/lignin biosorbents, chitin, kraft lignin, physicochemical properties, adsorption efficiency, hazardous metal removal

## Abstract

Novel, functional materials based on chitin of marine origin and lignin were prepared. The synthesized materials were subjected to physicochemical, dispersive-morphological and electrokinetic analysis. The results confirm the effectiveness of the proposed method of synthesis of functional chitin/lignin materials. Mechanism of chitin modification by lignin is based on formation of hydrogen bonds between chitin and lignin. Additionally, the chitin/lignin materials were studied from the perspective of waste water treatment. The synthetic method presented in this work shows an attractive and facile route for producing low-cost chitin/lignin biosorbents with high efficiency of nickel and cadmium adsorption (88.0% and 98.4%, respectively). The discovery of this facile method of synthesis of functional chitin/lignin materials will also have a significant impact on the problematic issue of the utilization of chitinous waste from the seafood industry, as well as lignin by-products from the pulp and paper industry.

## 1. Introduction

Recently, the growing problem of water pollution, especially with hazardous metals, has drawn the attention of specialists, who are trying to develop novel and relatively inexpensive methods for utilization of waste water. The methods generally used for this purpose include precipitation processes, membrane techniques, extraction and adsorption [1]. The latter one is considered the most effective and economical method of removing hazardous ions from aqueous solutions. In the recent years, multiple researchers have been focusing on the preparation of highly selective adsorbents of natural origin. The use of selective adsorbents that are common in the natural environment avoid generating additional pollution. The most commonly used natural adsorbents for removal of hazardous metals include straw, lignin, shells of invertebrates, peat, zeolites, fern, compounds contained in the structure of minerals, and microorganisms (bacteria, fungi, yeasts)—see for details [2,3,4,5]. Since it is very important to use effective and selective adsorbents for metal ion removal, in this report a unique system of chitin/lignin type was used as an adsorbent of Ni(II) and Cd(II) ions.

Lignin is one of the most widespread natural raw materials on earth. The structure of lignin is highly complex, and still has not been fully elucidated. In terms of chemistry, it is the most important to find a method of lignin acquisition that produces a compound without impurities. Methods of lignin separation from natural raw materials (mainly wood) involve its removal by dissolving, using appropriate chemical compositions based on inorganic materials or organic solvents in the presence of catalysts, and subsequent precipitation of the resulting lignin derivatives [6]. Such derivatives are called technical-grade lignin, regarded as waste. The complex chemical structure, valuable physicochemical properties and varied chemical composition of lignin have attracted the interest of scientists. It is biorenewable and inexpensive, which encourages research to use technical-grade lignin in the manufacture of materials with a significant added value. Formerly, more than 90% of the total production of technical-grade lignin was recycled for energy purposes in the production plants themselves, to recover any chemicals used for lignin digestion and improve the energy balance of the manufacturing process. There are a very limited number of reports stating that, when appropriately activated, lignin can be used in the manufacture of biomaterials [7], phenolic resins [8] biodegradable polymer compositions [9], active biosorbents [10,11,12,13], surfactants and dispersion agents [14] and in electrochemistry [15,16].

Of particular importance is the application of lignin as a potential adsorbent of hazardous metal ions. Excellent sorption properties of lignin were confirmed in the report by Guo *et al.* [11], among others. It was shown that lignin exhibits affinity to selected metal ions in the following order: Pb(II) > Cu(II) > Cd(II) > Zn(II) > Ni(II). Additionally, attention was paid to the two types of functional groups—carboxylic and phenolic—present on the surface of lignin. In another report, lignin’s sorption abilities were also examined [12]. In this case, the following order of affinity was established: Cr(VI) > Cd(II) > Cu(II) > Zn(II). In a paper by Mohan *et al.* [13], the superior ability of lignin over other biosorbents toward adsorption of copper and cadmium ions was reported. Those authors proposed a mechanism of adsorption and types of interactions between the biopolymer and the ions of the analyzed hazardous metals.

Chitin [poly(β-(1-4)-*N*-acetyl-d-glucosamine] is the second most abundant polysaccharide on Earth (see for review Ehrlich [17]), occurring in cell walls of fungi and diatoms [18], as well as in the exoskeletons of arthropods like crustaceans [19] and insects [20]. Recently, it was shown that it is also possible to isolate chitin from several marine [21,22,23] and freshwater [24,25] sponges. However, industrially chitin is obtained mainly from exoskeletons of shrimps and crabs, which are a seafood processing waste [26]. This waste is an emerging problem in countries like India, where the food industry is based mainly on seafood. Chitin represents an attractive alternative to other biomaterials because of its physicochemical characteristics, chemical stability, high reactivity, biodegradability, non-toxicity, and biocompatibility. All of these properties permit efficient chitinous waste management through the utilization of the biopolymer in various applications, including tissue engineering [27], drug delivery systems [28], catalysts [29] *etc.* Additionally, it was proven that chitin shows high intrinsic sorption affinity for dyes [30,31], and hazardous metal ions [32,33,34,35] which is an effect of the presence of one linear amino group per glucose ring, making electron pairs available for coordination [34,35]. The presence of functional (–OH, C=O and N–H) groups in the chitin molecule also enables efficient modification of chitin [31,36] to improve the separation performance of this low-cost and environmentally friendly adsorbent. Therefore, in this study it was decided to carry out a modification of chitin powder with kraft lignin to obtain functional low cost chitin/lignin sorbents with high efficiency of hazardous metal adsorption. A combination of these two polymers as metal ion adsorbents has not been previously studied, and in contrast to work formation of chitosan/lignin, our approach eliminates transformation of chitin to chitosan and therefore simplifies the synthesis procedure. It has been reported that chitin/lignin materials are effective in the sorption of hydrophobic organic contamination from water wastes [36].

## 2. Results and Discussion

### 2.1. Physicochemical Evaluation

#### 2.1.1. Morphological and Microstructure Characteristics

**Figure 1:**
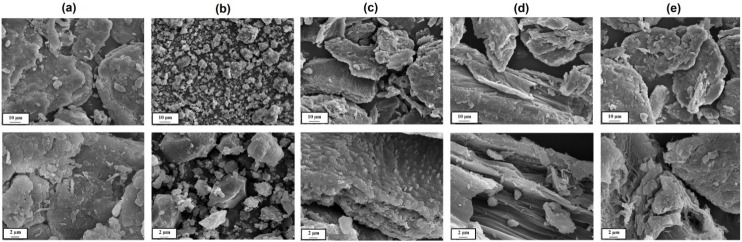
SEM images of (**a**) chitin; (**b**) kraft lignin; chitin/lignin materials labeled as (**c**) ChL 1; (**d**) ChL 4; (**e**) ChL 7 at different magnifications.

In Figure 1, microphotographs of pure α-chitin (Figure 1a) and lignin (Figure 1b) are shown. For a precise description of the morphological and microstructural character of the samples, pictures were taken at various magnifications. Chitin is characterized by a non-homogeneous structure, of which analysis indicates the presence of irregular particles with various shapes and sizes. In the structure of lignin, irregular shaped particles are also visible; however, its structure is more homogeneous and particles of smaller sizes can be observed (Figure 1b).

Additionally, in Figure 1, SEM images are presented at two different magnifications, taken for the selected chitin/lignin products. The microphotographs show a difference in the structure of the synthesized final products, in which the content of lignin is decreasing. From a morphological point of view, the photographs indicate that the ratio of the precursors used is crucial. In the prepared materials, chitin possesses fiber-like structures, different in shape and size, while lignin is characterized by individual and irregular particles of smaller size.

#### 2.1.2. FT-IR Spectroscopy

**Figure 2:**
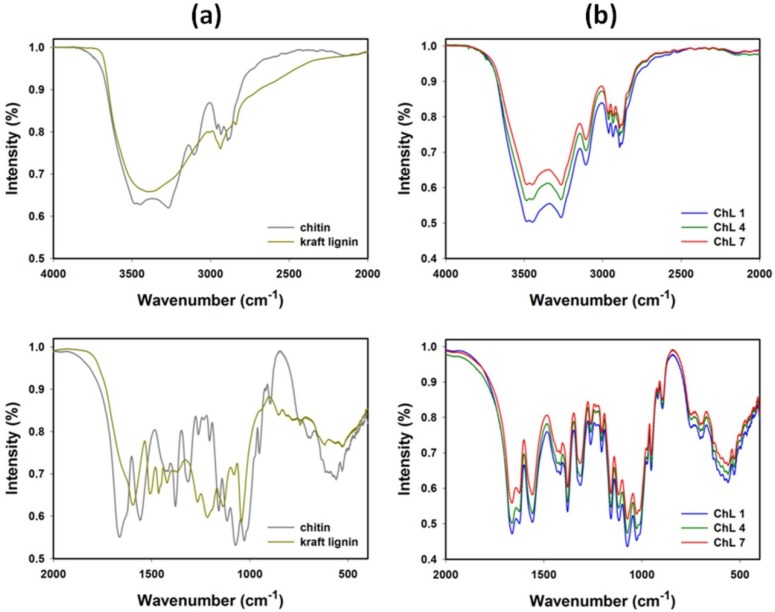
FT-IR analysis of precursors (**a**) and selected chitin/lignin materials (**b**).

Figure 2 shows the FT-IR spectra of chitin and lignin precursors (Figure 2a), and chitin/lignin hybrid materials (Figure 2b). Major bands are summarized in Table 1.

**Table 1:** Vibrational frequencies wavenumber (cm^−1^) attributed to chitin, kraft lignin, and chitin/lignin materials.

Chitin	Kraft lignin	Chitin/lignin material (ChL 1)	Vibrational assignment
3445	3387	3483	O–H stretching
3285	-	3264	N–H stretching
3107	-	3108	N–H stretching
2963	-	2966	CH_x_ stretching
2932	2935	2935	CH_x_ stretching
2875	-	2877	CH_x_ stretching
1663	-	1659	C=O (amide I) stretching
1630	1630	1624	C=O stretching
-	1595	-	C–C (aromatic skeleton) stretching
1558	-	1558	C–N (amide II) bending
-	1505	-	C–C (aromatic skeleton) stretching
-	1463	-	C–H, CH_3_ + CH_2_ bending
1430	-	1436	CH_2_ bending
**-**	1421	1415	C–C (aromatic skeleton) stretching
1378	-	1381	C–H bending
**-**	1370	-	O–H (phenolic OH) bending
**-**	1326	1328	C–O (syringyl unit) streching
1316	-	-	C–N (amide III) stretching
-	1266	-	C–O (guaiacyl unit) streching
1261	-	1259	N–H (amide III) bending
-	1216	-	C–OH (phenolic OH) stretching
1158	-	1156	C–O–C (ring), C–O stretching
-	1136	-	Aromatic C–H (guaiacyl unit), stretching
1116	-	1116	C–O–C (ring), C–O stretching
1073	-	1073	C–O–C (ring), C–O stretching
-	1040	-	C–OH + C–O–C (aliphatic OH + ether) stretching
1028	-	1028	C–O–C (ring), C–O stretching
951	-	951	CH_3_ bending
896	-	896	β-1,4-glycosidic bond
-	863	863	Aromatic C–H(guaiacyl unit), bending
-	745	745	Aromatic C–H(guaiacyl unit), bending
635	-	635	N–H bending

In the analysis of the spectrum of lignin the following bands were detected: stretching vibration bands of O–H groups (phenolic O–H and aliphatic O–H) at 3600–3200 cm^−1^, and C–H stretching vibrations at 2960–2920 cm^−1^ (CH_3_ and CH_2_). The wider band at 1710–1550 cm^−1^ results from the presence of C=O bond stretching vibrations. In the FT-IR spectrum of lignin, there are also significant bands with absorption maxima at the wavenumbers 1326 cm^−1^, 1266 cm^−1^ and 1216 cm^−1^, associated with stretching vibrations of C–O, C–O(H), and C–O(Ar) bonds of phenolic groups, as well as etheric bonds, which are important factors in connection of elements in the analyzed biopolymer. The presence of C–O–C etheric bonds is additionally confirmed by the stretching vibration band at 1040 cm^−1^. The last group of noteworthy characteristic bands of lignin consists of the in-plane deformation bands δ_ip_Ar C–H (1136 cm^−1^), and out-of-plane δ_op_Ar C–H (bands at wavenumbers lower than 1000 cm^−1^, including 863 cm^−1^, 745 cm^−1^). The present analysis of kraft lignin is in agreement with previously published data [37].

In turn, from the analysis of chitin spectrum, the following characteristic bands were found: a stretching vibration band of O–H groups at 3600–3400 cm^−1^, asymmetric stretching vibrations at 3285 cm^−1^, and symmetric vibrations at 3107 cm^−1^, attributed to N–H groups. The stretching vibration band in the range 3000–2800 cm^−1^ is associated with the presence of (CH_3_ + CH_2_) groups. Strong absorption bands at wavenumber 1663 cm^−1^ and 1630 cm^−1^ are associated with the presence of the amide I band, and correspond to stretching vibrations of C=O bonds. This splitting of the amide I band is characteristic for α-chitin, and stems from the occurrence of stretching vibrations of the intermolecular C=O···H–N and the intramolecular hydrogen bond C=O···H–OCH_2_. The presence of the absorption band at 1558 cm^−1^ in the spectrum of the isolated sample is attributed to the bending vibrations of the amide II band (N–H). The aforementioned band is undoubtedly associated with stretching vibrations of C–N. The region at 1430–1375 cm^−1^ is ascribed to bending vibrations associated with –CH_2_ and C–CH_3_ groups. A weak absorption band of stretching and bending vibrations associated with C–N and N–H groups (the so-called III amide band) appears at 1316 cm^−1^ and 1261 cm^−1^. A wide band at 1250–950 cm^−1^ is associated with asymmetric stretching vibrations of C–O–C groups and stretching vibrations of C–O groups. Of significance in the chitin spectrum is the presence of a characteristic band at 896 cm^−1^, which is attributed to the presence of β–1,4–glycosidic bonds in the biopolymer structure. The analysis carried out for chitin is in agreement with available literature data regarding α-chitin [21,38].

FT-IR spectra for selected chitin/lignin hybrid materials are presented in Figure 2b. Analysis of the spectra indicates that process of synthesis of chitin/lignin products was fully controlled, and completed with satisfactory results. Individual bands characteristic for the discussed precursors overlap with the bands obtained for the final products. Additionally, modification of the mass fraction of any precursor produces a change in the intensity of peaks. For instance, when the mass fraction of lignin in the products (from 1 to 7) decreases, the intensity of bands in the spectrum decreases as well. Also, the obtained spectra reveal shifts and deformations of the O–H stretching bands and I and II amide bands, which probably results from hydrogen bond formation between chitin and lignin.

#### 2.1.3. XPS Analysis

The surface composition of samples of α-chitin, lignin and chitin/lignin biosorbent (ChL 1) was examined by means of X-ray photoelectron spectroscopy. The elemental compositions calculated from survey spectra in the binding energy range 0–1000 eV are given in Table 2.

**Table 2:** Elemental composition of the surface of examined samples as calculated by XPS analysis.

Sample	C	O	N	Na	S	Ca	Cl
	at. %						
ChL 1	64.8	29.5	4.8	-	-	0.9	-
chitin	60.0	32.9	5.9	0.3	-	0.6	0.3
kraft lignin	68.0	25.0	-	5.0	2.0	-	-

The lignin sample is contaminated with sodium and sulfur, presumably resulting from its preparation in the kraft process, as observed previously elsewhere [39]. On the surface of chitin and ChL 1 samples, some traces of calcium and chlorine were detected, presumably due to environmental contamination.

Carbon, nitrogen and oxygen atoms are the main components of both chitin and ChL 1 samples, while lignin does not contain nitrogen. The ratio of oxygen to carbon for chitin is almost 0.5, as reported before [40]. The O/C ratio calculated for lignin is 0.36, relatively close to the theoretical value for lignin given by Johansson *et al.* [41] as 0.34. The ratio of oxygen to carbon for the ChL 1 sample lies between the values for chitin and lignin, at 0.45.

**Figure 3:**
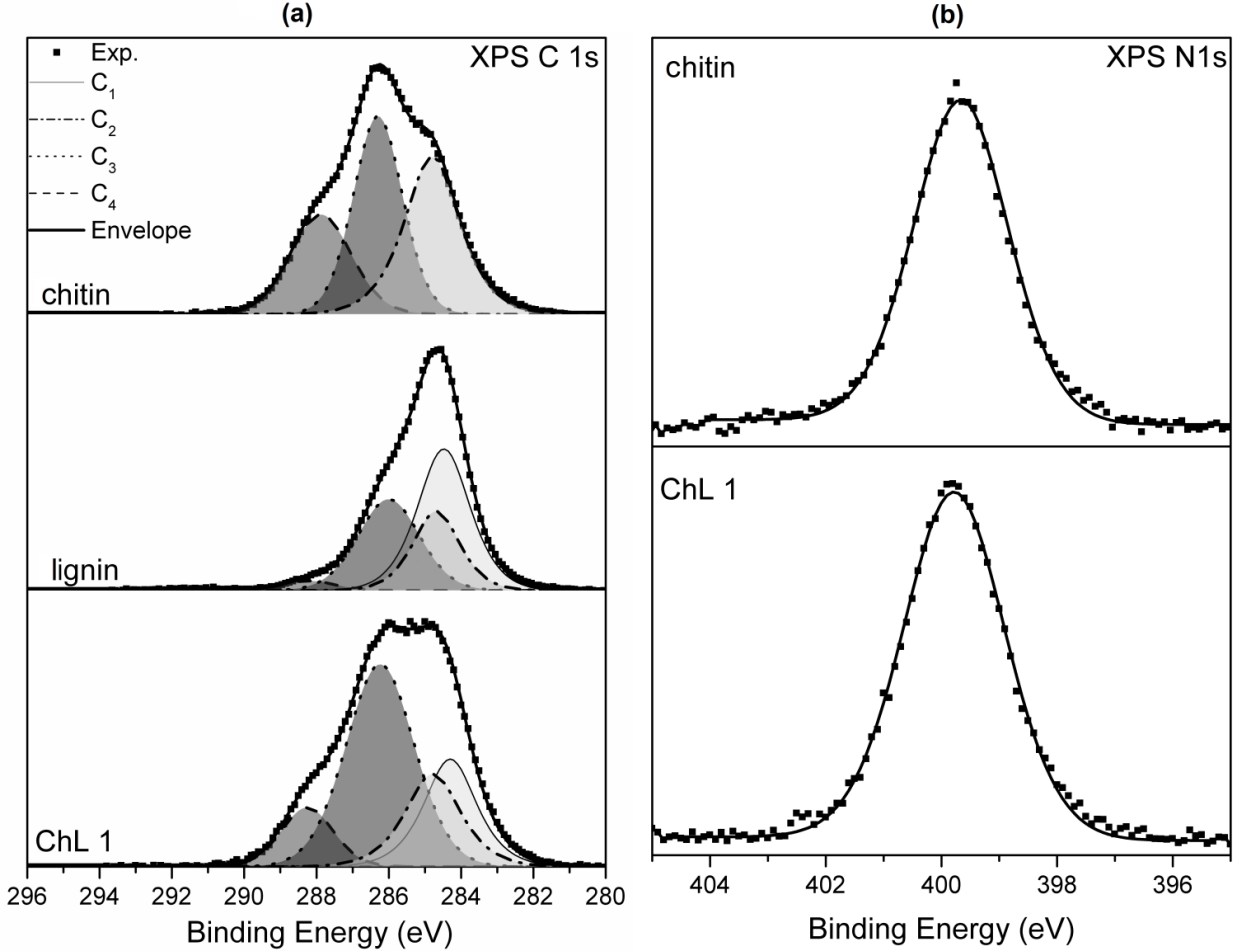
(**a**) XPS C 1s spectra for chitin, kraft lignin and ChL 1 samples. The assignment of components C1–C4 is described in the text; (**b**) XPS N 1s spectra for chitin and ChL 1.

The XPS C 1s peak was examined in detail for all analyzed samples. The acquired spectra have a relatively complex profile (see Figure 3a).

Deconvolution of the experimental data was performed using a model consisting of four basic components of C 1s transition: C_1_–C_4_. Component C_1_, having a binding energy of 284.5 ± 0.1 eV, corresponds essentially to non-functionalized carbon atoms located in aromatic rings expected in the lignin structure. Component C_2_, having a binding energy of 284.8 eV, is attributed to all other non-functionalized sp^2^ and sp^3^ carbon atoms, bonded either with a second carbon or with hydrogen atoms. Component C_3_, shifted 1.4 ± 0.2 eV from component C_2_ in the direction of increasing binding energies, is ascribed to a group of differently bonded carbon atoms linked to one atom of oxygen or nitrogen. The group comprises the following functional groups presumably present in the studied materials: C–O–C, C–OH, C–N–C and C–NH_2_. The carbon denoted by the asterisk in the *C–O–C=O group can also contribute to the signal of component C_3_. Component C_4_, shifted 2.9 ± 0.2 eV from component C_2_ in the direction of increasing binding energies, also corresponds to a set of functional groups: C=O, O–C–O, N–C–O and N–C=O. The binding energy assignments described above are based on the energy shifts given in Appendix E of the reference paper [42]. The relative surface functional group compositions obtained from the decomposition of the C 1s signal are given in Table 3. The total C 1s intensity is taken as 100.

**Table 3:** Distribution of functional groups calculated on the basis of the deconvolution model of XPS C 1s peak.

Sample	C_1_	C_2_	C_3_	C_4_
ChL1	21	19	49	11
chitin	-	37	39	24
kraft lignin	43	22	32	3

An expected component ratio for pure chitin is C_2_:C_3_:C_4_ = 25:50:25 [43]. In the present study, there is a substantial excess of non-functionalized carbon atoms (component C_2_) and the profile of the XPS C 1s peak rather resembles a modified chitin structure [40,44]. The reason for the increased concentration of carbon atoms linked only with other carbon atoms or hydrogen is either decomposition of oxygen-bearing functional groups, or surface contamination by adventitious carbon.

The shape of the XPS C 1s peak for lignin is relatively close to the theoretical one [45]. The ratio of (C_1_ + C_2_):C_3_:C_4_ = 65:32:3 calculated for the present data is in good agreement with that observed elsewhere [46] where the corresponding ratio is given as (C_1_ + C_2_):C_3_:C_4_ = 65:29:3.

The XPS spectrum of the ChL 1 sample, which is a product originating from chitin and lignin samples, has a complex structure. Assuming that this material contains functional groups coming from both substrates, all four carbon components C_1_–C_4_ were used during the curve-fitting procedure. The peak fitting indicates that the ChL 1 sample contains approximately equal numbers of aromatic and aliphatic non-functionalized carbon atoms (components C_1_ and C_2_, respectively). Component C_3_ represents about one half of all carbon atoms present in the material, demonstrating the abundance of C–OH and C–O–C groups, likely with a contribution from C–N bonds.

The XPS N 1s peak was additionally examined in detail to confirm the chemical state of nitrogen atoms present in the chitin and ChL 1 samples (see Figure 3b). The observed profiles of N 1s transition are virtually identical for both materials. They are both symmetrical with mixed Gaussian-Lorentzian profile shape, and with a maximum at a binding energy of 399.8 eV. It is assumed that they represent one chemical state of the nitrogen atoms. There is a disagreement concerning the attribution of chemical bonds of nitrogen to the experimentally observed XPS N 1s peaks of chitin. Oh *et al.* [44] attributed a component at binding energy 401.4 eV observed in the spectrum of regenerated chitin to the nitrogen of acetyl amide group, while a component at 399.6 eV was ascribed to the amine group. However, the nitrogen chemical environment representative of the acetyl amide group is usually matched with the N 1s component around 400.0 eV [47]. Therefore, the N 1s peak observed here is attributed to the acetyl amide groups which are a part of the chitin structure.

#### 2.1.4.^13^C CP MAS NMR Spectroscopy

**Figure 4:**
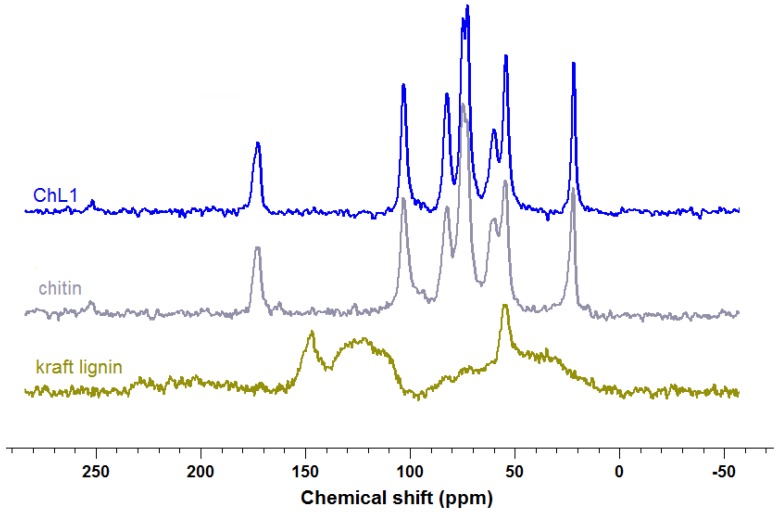
^13^C CP MAS NMR spectra of chitin, lignin, and ChL 1 material.

Figure 4 shows the ^13^C CP MAS NMR spectra of the pure precursors’ chitin and kraft lignin, and of the hybrid material obtained by the reaction of chitin and lignin in mass ratio of 1:1.

Detailed analysis of the signals, assigned to specific, carbon-containing functional groups, is presented in Table 4.

The ^13^C CP MAS NMR spectrum of the chitin before modification exhibits characteristic narrow and intense signals for α-chitin [38,48], while for lignin broad lower intensity signals are present [49]. Modification of chitin with lignin obviously results in characteristic changes in the spectrum. Particularly important is the fact that in the case of the analyzed hybrid material ChL 1, there was recorded an increase in the characteristic chemical shifts in comparison to chitin prior to modification. Especially the C6 (59.94 ppm) and C3 (73.08 ppm) signals are stronger in the hybrid material. Also observable are slight chemical shifts which confirm the hypothesis that chitin and lignin are connected via hydrogen bonds. The formation of hydrogen bonds is possible due to the significant amount of various functional groups present in the compounds, especially in kraft lignin. Analysis of the ^13^C CP MAS NMR spectra fully confirmed the effectiveness of formation of the chitin/lignin material.

**Table 4:** The chemical shift value (δ, ppm) of ^13^C CP MAS NMR spectrum of chitin, kraft lignin, and ChL 1 sample.

Chitin	Lignin	ChL 1	Assignment
-	13.6	-	γ–CH_3_ in n-propyl side chain
22.1	-	21.9	CH_3_ in acetamide group
-	24.3	-	CH_3_ or CH_2_ group in saturated side chains
-	36.1	-	CH_3_ groups, ketones (conj.) or in aliphatic
-	52-54	54.2	C-β in β-5 and β-β units
54.4	-	54.2	C2 in hexose ring
-	55.6	-	C in Ar–OCH_3_
59.6	-	59.9	C6 in hexose ring
73.1	-	72.6	C3 in hexose ring
74.7	-	74.7	C5 in hexose ring
-	74-1	72.6	C-α in guaiacyl type β-0-4 units (*threo* and *erythro*) C-γ in β-β, C-γ, β-aryl ether
82.2	-	82.4	C4 in hexose ring
-	85-83	-	C-β in guaiacyl type β-0-4 units (*threo* and *erythro*)
103.3	-	103.1	C1 in hexose ring
-	112-110	-	C-2 in guaiacyl units
-	117-113	-	C-5 in guaiacyl units
-	118-119	-	C-6 in guaiacyl units
-	121.4	-	C1 and C6 in Ar–C(=O)C–C
-	128.2	-	C-α and C-β in Ar–CH=CH–CH_2_OH
-	129.3	-	C-α and C-β in Ar–CH=CH–CHO
-	143.3	-	C-4 in ring B of β-5 units, C-4/C-4′ of non–etherified 5-5 units
-	145.8	145.5	C-4 in non-etherified G units
-	146.2	-	C-3 in non-etherified G units (β-0-4 type)
-	146.8	-	C-4 in etherified G units
-	169-172	-	C=O in φ–COOH, Ester C=O in φ–C(=O)OR and R–C(=O)OCH_3_
173.4	-	172.7	C=O in acetamide group
-	192-202	-	C=O in φ–CH=CH–CHO, C=O in φ–C(=O)CH(–O φ)–C– and other carbonyl groups

#### 2.1.5. Elemental Analysis

Table 5 contains results from elemental analysis describing the content of nitrogen, carbon, hydrogen and sulfur in the prepared chitin/lignin materials as well as in the pure precursors.

In the case of pure chitin, the carbon content is 40.54%, while the hydrogen content is 7.36%. Nitrogen is also present in the structure of chitin, accounting for about 6.21% of the sample by mass; this is associated with *N*-acetylglucosamine units (to be precise, 2-(acetylamino)-2-deoxy-d-glucose). Kraft lignin also has carbon and hydrogen in its structure (42.21% and 5.02%, respectively), but additionally sulfur is found (3.14%). The presence of sulfur can be explained by the process of separation of cellulose from lignin via sulfuric wood digestion, the so-called kraft process.

Analysis of the results for the chitin/lignin materials proves the diversity of elemental composition of the products, and intermediately confirms the effectiveness of the preparation method used. The percentage content of nitrogen in the respective samples (ChL 1 to ChL 7) is similar, which reflects the constant quantity of chitin used for preparation of the products. The content of remaining elements in the final products depends strictly on the type of sample, and thus on the quantities of the precursors used. With decreasing content of lignin in the final products, the percentage content of carbon, hydrogen and sulfur also decreases. As far as sulfur is concerned, its decreasing content serves as evidence for the presence of the element in the structure of lignin and lignocellulose materials.

**Table 5:** Content of the examined elements in the precursors and chitin/lignin materials.

Sample name	Elemental content (%)			
	N	C	H	S
ChL 1	6.01	44.17	8.37	1.16
ChL 2	6.03	44.04	8.31	0.96
ChL 3	6.03	44.01	8.27	0.79
ChL 4	6.03	43.93	8.24	0.63
ChL 5	6.01	43.75	8.23	0.49
ChL 6	6.03	43.72	8.20	0.27
ChL 7	6.02	43.58	8.19	0.06
chitin	6.21	40.54	7.36	-
kraft lignin	-	42.21	5.02	3.14

#### 2.1.6. Electrokinetic Characteristics

A very important factor in the applicability of new materials is electrokinetic behavior. Therefore, in the next stage of the experiment, values of zeta potential at selected pH were measured, and the isoelectric point (pH_IEP_) was determined. The obtained results are presented in Table 6. Determination of the zeta potential of the discussed compounds enabled indirect confirmation of the effectiveness of the suggested synthesis method.

**Table 6:** Summary of the zeta potential of chitin/lignin materials, and the pure precursors at the selected pH.

Sample name	Zeta potential (mV) *vs.* pH						pH_IEP_
	2	4	6	8	10	12	
ChL 1	−1.3	−17.5	−26.5	−37.0	−43.2	−46.3	1.8
ChL 2	1.7	−14.2	−24.2	−35.0	−41.0	−43.1	2.2
ChL 3	2.1	−11.1	−22.0	−33.9	−39.0	−42.0	2.7
ChL 4	3.9	−10.0	−20.9	−30.1	−37.9	−40.9	2.7
ChL 5	5.2	−8.0	−19.5	−27.9	−36.5	−38.2	2.8
ChL 6	7.1	−7.5	−17.3	−23.0	−34.2	−37.9	3.1
ChL 7	9.3	−6.5	−15.1	−20.5	−31.9	−36.0	3.4
chitin	19.9	−8.5	−23.6	−31.4	−37.2	−40.1	3.5
kraft lignin	−20.1	−35.2	−40.2	−43.8	−48.3	−51.2	-

In the first stage of the experiment, zeta potentials of chitin and kraft lignin were determined in order to measure the electrokinetic properties of the precursors. From the electrokinetic analysis of chitin, it is found that the biopolymer exhibits negative values of zeta potential over a wide pH range. Due to the fact that at very low and very high pH values the measuring error increases, the range of measurement was limited to pH values from 2 to 12. The measured values of zeta potential of the biopolymer strongly depend on the pH. Moreover, chitin is found to have a pH_IEP_ of 3.5. This is caused by dissociation of –NH_2_ groups, which are always present in the structure of chitin. These groups play a very important role in changes of surface properties. Large quantities of H^+^ ions induce ionization of –NH_3_^+^ groups, and as a result a positive charge is formed on the surface of chitin. This is called a protonation effect. With an increase in the quantity of H^+^ ions, the dissociation process is limited and zeta potential decreases [50]. Chitin is most electrokinetically stable in aqueous solutions at pH 8–12. Kraft lignin exhibits negative values of zeta potential over the whole analyzed pH range, and does not reach the pH_IEP_ value. The biopolymer is characterized by excellent electrokinetic stability regardless of the pH value. Detailed information regarding the electrokinetic properties of kraft lignin can be found in a previous publication [37].

In the next stage of the experiment, electrokinetic properties of the chitin/lignin materials were determined. From the measured values of zeta potential it can be concluded that the mass contribution of the biopolymers in the composite influences its electrokinetic properties. In the case of a composite with equal amounts of both biopolymers, dominant electrostatic interactions originating from lignin can be observed, which results from the presence of functional groups on its surface. However, the obtained values of electrokinetic potential are lower than in the case of pure lignin. This phenomenon is caused by interactions of amine groups originating from chitin. Analysis of the systems shows that as the mass contribution of lignin decreases, the observed protonation effect becomes increasingly visible. This is also reflected by the values found for the isoelectric point.

The results confirm the effectiveness of the proposed method of synthesis of the discussed composites. What is more, the satisfactory results support the belief that the examined systems will find wide application in advanced industrial methods where the electrokinetic stability of aqueous dispersions plays an important role.

#### 2.1.7. Thermal Stability

**Figure 5:**
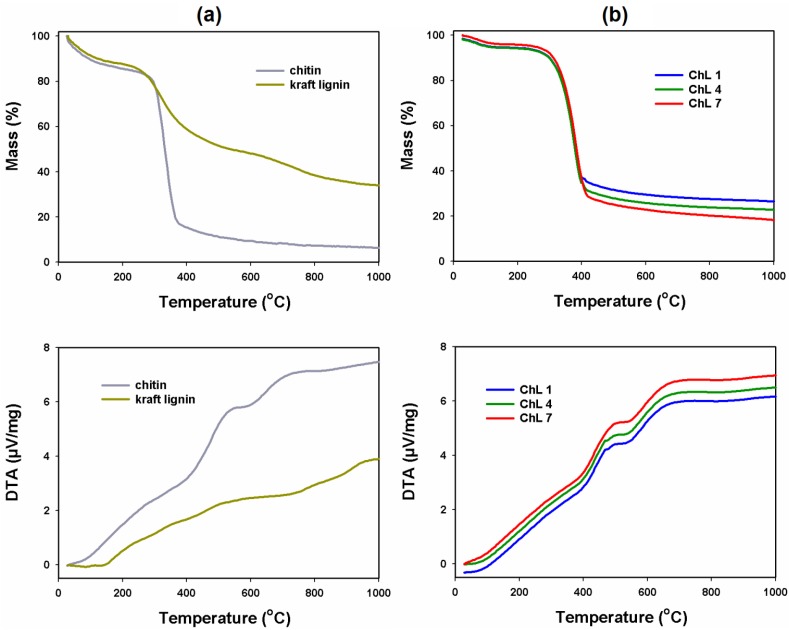
TG/DTA analysis of chitin and kraft lignin (**a**) and three selected chitin/lignin materials (**b**).

The thermal behavior of chitin, kraft lignin (Figure 5a) and chitin/lignin hybrid materials (Figure 5b) were determined using TG and DTA.

The results of TG/DTA analysis obtained for chitin indicate two degradation steps. The first one occurs below 200 °C and is associated with endothermic desorption of physically bound water. The second begins at 280 °C, and is attributed to the endothermic thermal degradation of α-chitin, which is primarily a result of the single-step reaction of depolymerization of the chitin molecular structure, including dehydration of polysaccharide rings and formation of low volatile products and char [51]. The TG curve of lignin indicates that there exist two distinct weight loss stages during the pyrolysis of this biopolymer. The first step is mainly caused by the release of water. According to the results reported by Liu *et al.* [52], with the use of a TG-FTIR combined technique it is possible to make a detailed interpretation of each stage of lignin pyrolysis. Above a temperature of 100 °C, a process of cracking of aliphatic hydroxyl groups in the lateral chains also occurs, which generates water and CO_2_ due to breakage of lateral C–C bonds. The main exothermic degradation step occurs in the range 250–570 °C. According to the literature [52,53], this step is divided into three stages associated with the release of various volatile compounds. Phenolic compounds containing aromatic ring, hydroxyl and alkyl groups are released at ~270 °C. Release of methanol due to the reaction of hydrogenation of the methoxy groups (–OCH_3_) in the aromatic ring starts at about 380 °C. Finally, at 530 °C, formation of secondary methane begins, probably generated from secondary cracking of the primary compounds [54].

In the case of the chitin/lignin hybrid materials we observed two degradation steps. The first one, below 200 °C, is associated with endothermic desorption of water. The second degradation step begins at 270 °C and is attributed to endothermic thermal degradation of chitin. However, the observed endothermic effect is lower than in the case of pure chitin, and decreases with an increase of lignin content in the chitin/lignin materials. This phenomenon can be explained by the exothermic degradation of lignin: part of the energy emitted from the degradation of lignin is consumed in the degradation of the biopolymer; hence, the observed endothermic effect is lower for the composites. Additionally, it is important to note that the thermal degradation of chitin results in a higher amount of residue than the same process for pure lignin.

#### 2.1.8. Porous Structure Properties

**Figure 6:**
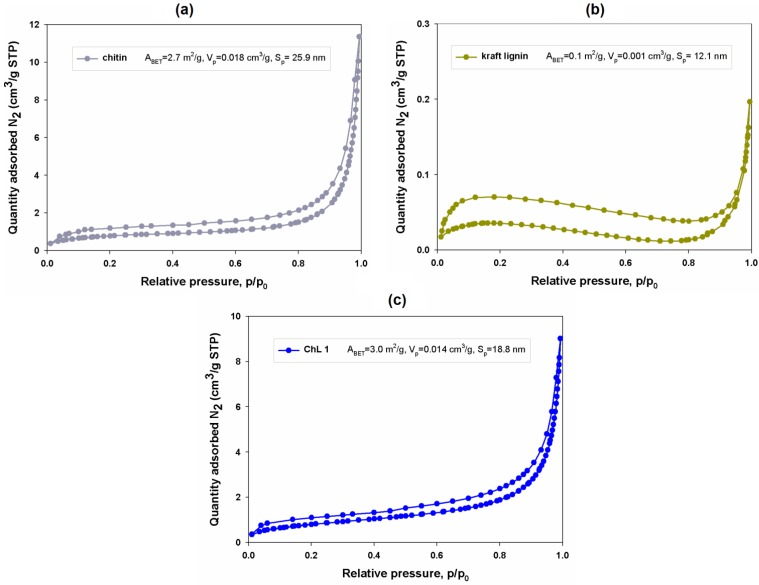
Nitrogen adsorption/desorption isotherms and porous structure parameters for chitin (**a**); kraft lignin (**b**); and the chitin/lignin material labeled as ChL 1 (**c**).

For determination of the sorption properties of the materials, it is extremely important to analyze their porous structure. For this purpose, adsorption/desorption isotherms were determined, and BET surface area, total pore volume and size of pores were calculated. The results are shown in Figure 6.

The biopolymers used in the experiment are characterized by a low value of BET surface area. For chitin *A_BET_* = 2.7 m^2^/g, and for lignin the value is even lower: *A_BET_* = 0.1 m^2^/g. Values of total volume of pores and size of pores are higher for chitin, at *V_p_* = 0.018 cm^3^/g and *S_p_* = 25.9 nm. By comparison, lignin has lower values: *V_p_* = 0.001 cm^3^/g and *S_p_* = 12.1 nm. Despite the low value of BET surface area, the materials can still be treated as effective and selective sorbents of hazardous metal ions and harmful organic compounds. In the case of this group of materials, an important role in the process of hazardous metal ion adsorption is played by acidic functional groups (particularly hydroxyl groups originating from phenol and carboxylic groups). The presence of these functional groups is confirmed by the FT-IR spectra presented earlier. Similarly, electrokinetic analysis suggests that the analyzed samples possess negative surface charge originating from organic compounds. The negative value of the surface charge results from dissociation of H^+^ ions coming from acidic functional groups. These properties have a great impact on the adsorption of positively charged metal ions, as has been confirmed in other studies [54].

In order to carry out further analysis concerning the adsorption of nickel(II) and cadmium(II) ions on the surface of chitin/lignin materials, the sample labeled as ChL 1 was used. This material has optimal physicochemical and electrokinetic properties which might influence the effectiveness of the process of removal of nickel(II) and cadmium(II) ions from model aqueous solutions. This material contains the largest quantity of functional groups, which might be important in the process of heavy metal ion adsorption. The tested sample was prepared with the ratio of precursors at 1:1. For material ChL 1 the surface area is 3.0 m^2^/g, the total pore volume is *V_p_* = 0.014 cm^3^/g, and the pore size is *S_p_* = 18.8 nm.

### 2.2. Batch Adsorption Study

#### 2.2.1. Effect of Contact Time on Sorption Efficiency

**Figure 7:**
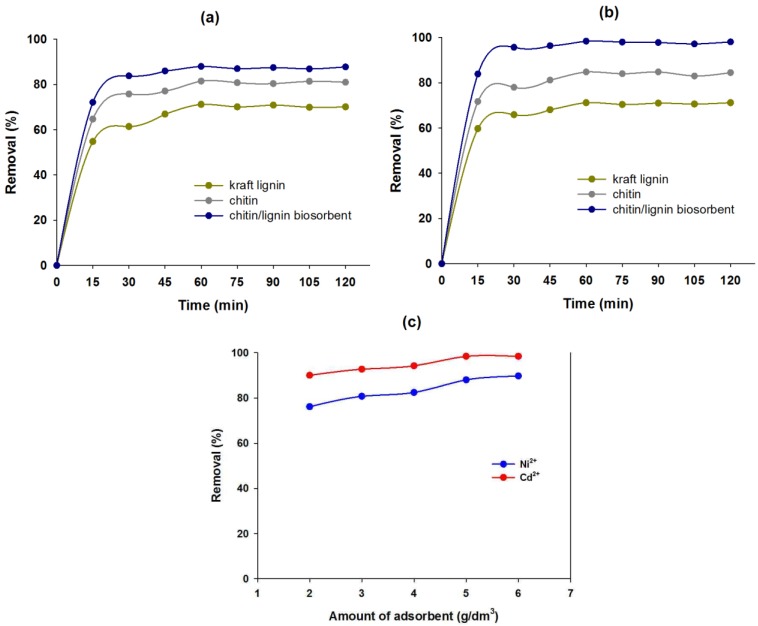
Effect of contact time on (**a**) nickel(II); (**b**) cadmium(II) removal by chitin, kraft lignin and chitin/lignin biosorbent; (**c**) influence of quantity of chitin/lignin material on nickel(II) and cadmium(II) removal efficiency (pH = 7 and temperature 25 °C).

The prepared chitin/lignin biosorbent has unique physicochemical and electrokinetic properties which enables it to be used as an effective sorbent of hazardous metal ions. In this work, preliminary tests of nickel(II) and cadmium(II) ion removal from aqueous solution were performed, using the chitin/lignin sorbent as well as pure chitin and lignin. The influence of time (15–120 min) on the effectiveness of nickel(II) and cadmium(II) ion adsorption (30 mg/dm^3^) was examined. Example data from the experiment are given in Figure 7a,b. From the results, it can be concluded that the adsorption equilibrium, for the chitin/lignin biosorbent as well as pure biopolymers, is reached after 60 min, which can be considered the most efficient adsorption time.

It is noteworthy that in the removal of nickel(II) and cadmium(II) ions, significantly higher values for the adsorption process were achieved for the chitin/lignin sorbent than in the case of the pure precursors. This indicates the higher affinity of the surface of chitin/lignin sorbent to cadmium(II) ions in comparison with the sole precursors. From analysis of the results, it is noted that the adsorption efficiency on the surface of chitin/lignin sorbent was significantly higher in the case of cadmium(II) adsorption (83.9%–98.4%) than in the case of nickel(II) (72.1%–88.0%). This observation indicates the greater affinity of the sorbent for adsorption of cadmium(II) ions than nickel(II).

Analyzing the results it can be stated that the highest amount of adsorbed nickel(II) and cadmium(II) ions (at equilibrium) was noted in the case of chitin/lignin biosorbents (5.28 mg(Ni^2+^)/g and 5.90 mg(Cd^2+^)/g). The observation points to better sorption abilities of metal ions using this novel, functional material than in the case of pure lignin and chitin precursors (see Table 7). Noteworthy is also the fact that the surface of the biosorbent shows higher affinity to cadmium(II) ions.

#### 2.2.2. Effect of Quantity of Chitin/Lignin Biosorbents on Sorption Efficiency

The quantity of used sorbent also influences the effectiveness of nickel(II) and cadmium(II) ion adsorption. This is shown by the analysis presented in Figure 7c.

**Table 7:** The amount of metal ions adsorbed at equilibrium (*q_e_*) for kraft lignin, chitin and chitin/lignin biosorbents.

Kind of sorbents	The amount of metal ions adsorbed at equilibrium (mg/g)	
	Ni^2+^	Cd^2+^
lignin	4.27	4.28
chitin	4.89	5.09
chitin/lignin biosorbents	5.28	5.90

The adsorption process was performed over 60 min from model solutions with a metal ion concentration of 30 mg/dm^3^, using varying amounts of the sorbent (2–6 g/dm^3^). The effectiveness of the removal of nickel(II) and cadmium(II) ions increases with increasing content of the sorbent, which is a result of the larger surface area of the adsorbent. The highest removal efficiency of ions was achieved for 5 g/dm^3^ of the sorbent, similarly for model solutions of both hazardous metal ions. It should be noted that, analogously to the previous results, the chitin/lignin biosorbent exhibits a higher affinity for cadmium(II) ions (removal efficiency 90.0%–98.5%) than for nickel(II) (removal efficiency 76.1%–89.7%).

The results obtained undoubtedly demonstrate the effectiveness of the process of nickel(II) and cadmium(II) ion adsorption from model aqueous solutions with the use of chitin/lignin biosorbents. Although these are only preliminary results, the sorption abilities of the chitin/lignin materials have already been shown to be worthy of attention.

## 3. Experimental Section

### 3.1. Materials

α-Chitin powder from crab shells (technical grade, Sigma-Aldrich, Munich, Germany) was combined with kraft lignin (reagent grade, Sigma-Aldrich). In order to obtain final products in the form of chitin/lignin materials, various reagent fractions were used, which were additionally treated with 15% hydrogen peroxide (Chempur, Piekary Śląskie, Poland). The adsorption process was carried out with use of selected inorganic salts: nickel(II) nitrate hexahydrate and cadmium(II) nitrate tetrahydrate were used (Sigma-Aldrich).

### 3.2. Preparation of Chitin/Lignin Materials

Preparation of the chitin/lignin products began with soaking of the required quantity of lignin in 100 cm^3^ of 15% hydrogen peroxide (lignin activation).

The system was stirred for about 30 min using a high speed stirrer (EUROSTAR digital IKA Werke GmbH, Staufen, Germany) with a mixing rate of 1000 rpm. A suitable quantity of chitin was then added to the solution of activated lignin, and the mixture underwent vigorous mixing for about 2 h. The obtained chitin/lignin final material was filtered under reduced pressure and washed with distilled water. Next the product was dried in a convectional dryer (Memmert, Munich, Germany) at a temperature of 105 °C (for about 24 h). In this way, seven systems, differing in precursor content, were prepared. Details of the quantities of precursors used are given in Table 8.

**Table 8:** List of chitin/lignin materials obtained, with specific amounts of precursors used.

Sample name	The weight ratio of precursors (chitin:lignin)	Amount of H_2_O_2_ (cm^3^)
ChL 1	1:1	100
ChL 2	1:0.75	
ChL 3	1:0.5	
ChL 4	1:0.3	
ChL 5	1:0.2	
ChL 6	1:0.1	
ChL 7	1:0.05	

### 3.3. Physicochemical Evaluation

The surface morphology and microstructure of the chitin/lignin products were examined on the basis of SEM images recorded from an EVO40 scanning electron microscope (Zeiss, Jena, Germany). Prior to the testing, the samples were coated with Au for a time of 5 s using a Balzers PV205P coater (Oerlikon Balzers Coating SA, Brügg, Switzerland).

The presence of expected functional groups was confirmed by Fourier transform infrared (FT-IR) spectroscopy, recorded on an EQUINOX 55 spectrophotometer (Bruker, Karlsruhe, Germany). Here, the materials were analyzed in the form of tablets, made by pressing a mixture of anhydrous KBr (*ca.* 0.1 g) and 1 mg of the tested substance in a special steel ring under a pressure of approximately 10 MPa. The investigation was performed at a resolution of 0.5 cm^−1^.

X-ray photoelectron spectra (XPS) were obtained using Al Kα (*hν* = 1486.6 eV) radiation with a Prevac system equipped with a Scienta SES 2002 electron energy analyzer (VG Scienta, St. Leonards-on-Sea, UK) operating at constant transmission energy (Ep = 50 eV). The spectrometer was calibrated using the following photoemission lines (with reference to the Fermi level): EB Cu 2p_3/2_ = 932.8 eV, EB Ag 3d_5/2_ = 368.3 eV and EB Au 4f_7/2_ = 84.0 eV. The instrumental resolution, in terms of the full width at half maximum (FWHM) of the Ag 3d_5/2_ peak, was 1.0 eV. The samples were loosely placed into a grooved molybdenum sample holder. The analysis chamber was evacuated during the experiments to better than 1 × 10^−9^ mbar.

Data processing involved background subtraction by means of “S-type” integral profile and a curve-fitting procedure (a mixed Gaussian-Lorentzian function was employed) based on a least-squares method (CasaXPS software, SurfaceSpectra Ltd., Manchester, UK). Experimental errors were estimated to be ±0.2 eV for the photoelectron peaks of carbon and nitrogen. Charging effects were corrected using the C 1s component ascribed after deconvolution to the aliphatic carbon bindings (component C_2_) and taken to be 284.8 eV. The reproducibility of the peak position thus obtained was ±0.2 eV. The surface composition of the samples was obtained on the basis of the peak area intensities of the C 1s, O 1s, N 1s, Na 1s, S 2p, Ca 2p and Cl 2p transitions using the sensitivity factor approach and assuming homogeneous distribution of elements in the surface layer.

^13^C CP MAS NMR measurement was carried out on a DSX spectrometer (Bruker). For the determination of NMR spectra, a sample of about 100 mg was placed in a ZrO_2_ rotator with diameter 4 mm, which enabled spinning of the sample. Centrifugation at the magic angle was performed at a spinning frequency of 8 kHz. The ^13^C CP MAS NMR spectra were recorded at 100.63 MHz in a standard 4 mm MAS probe using a single pulse excitation with high power proton decoupling (pulse repetition 10 s, spinning speed 8 kHz).

The elemental contents of the products were established with the use of a Vario EL Cube instrument made by Elementar Analysensysteme GmbH, which is capable of registering the percentage content of carbon, hydrogen, nitrogen and sulfur within samples, after high-temperature combustion. A properly weighed sample was placed in an 80-position autosampler and subjected to combustion. The decomposed sample was transferred in a stream of helium gas into an adsorption column, where the percentage of each element was analyzed. The results are given to ±0.01%, and each is obtained by averaging three measurements.

Zeta potential was measured by the electrophoretic light scattering method using a Zetasizer Nano ZS instrument equipped with an autotitrator (Malvern Instruments Ltd., Worcestershire, UK). The zeta potential was determined over a pH range of 2–10, using 0.001 M NaCl solution. Before the measurement was performed, the analyzed dispersions were stabilized for 15 min in an ultrasonic bath. To avoid possible measurement errors, every sample was measured three times, and the mean value and standard deviation were calculated. The standard deviation of the zeta potential at a given pH was ±1.5 mV or less, and the error in the pH was estimated to be 0.02 pH units or lower.

Thermogravimetric (TG) and differential thermal analyses (DTA) of chitin/lignin products were carried out with a Jupiter STA 449F3 analyzer (Netzsch, Selb, Germany) with an Al_2_O_3_ crucible. The measurements were performed in a nitrogen atmosphere at a heating rate of 10 °C min^−1^. The samples were heated up to 1000 °C, starting from 25 °C.

In order to characterize the properties of the porous structure, nitrogen adsorption/desorption isotherms, and parameters such as surface area (*A_BET_*), total volume of pores (*V_p_*), and mean size of pores (*Sp*), were determined using an ASAP 2020 instrument (Micromeritics Instrument Co., Norcross, GA, USA). All samples were degassed at 80 °C for 4 h prior to measurement. The surface area was determined by the multipoint Brunauer-Emmett-Teller method using the adsorption data as a function of relative pressure (*p*/*p*_0_). The Barrett-Joyner-Halenda algorithm was also applied to determine the total volume of pores and mean pore size.

### 3.4. Adsorption Experiments

In order to determine the optimal time of metal ion removal from model aqueous solutions, the adsorption process was carried out over a range of time periods: 15, 30, 45, 60, 75, 90, 105 and 120 min for a nickel(II) and cadmium(II) ion concentration of 30 mg/dm^3^ (pH = 7 and temperature 25 °C). The adsorption time optimization was performed independently for three types of sorbents: pure lignin, pure chitin, and final chitin/lignin biosorbent with a component ratio of 1:1.

The amount of metal ions adsorbed at equilibrium, *q_e_* (mg/g), was calculated by the following relationship (1):

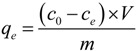
(1)
where *q_e_* is the amount of metal ions adsorbed at equilibrium (mg/g), *C*_0_ is the initial concentration of metal ions (mg/L), *C_e_* is the equilibrium metal ions concentration (mg/L), *V* is the volume of solution (L) and *m* is the mass of the biosorbents (g). The prepared solutions of nickel(II) and cadmium(II) ions were placed in conical flasks. To the solution, 5.0 g/dm^3^ of adsorbent was added. The system was stirred using a magnetic stirrer (IKA Werke GmbH) for a set time. At the appropriate time, the obtained mixture was filtered. Each filtrate was analyzed to measure the effectiveness of the adsorption process.

An important element of the experiment was to determine the influence of the quantity of the sorbent on the effectiveness of removal of nickel(II) and cadmium(II) from aqueous solutions. For this purpose, the adsorption process of nickel and cadmium ions in solutions of concentration 30 mg/dm^3^ was carried out using various amounts of chitin/lignin material (2–6 g/dm^3^) for 60 min. Optimization of the mass of the sorbents was performed solely for model solutions of nickel(II) and cadmium(II). In order to determine the effectiveness of nickel(II) and cadmium(II) ion removal, AAS analysis was performed (Z-8200 spectrometer, Hitachi, Tokyo, Japan). The results obtained from the analysis were used to calculate the efficiency of the process.

## 4. Conclusions

In this report, a new method for the preparation of novel and functional chitin/lignin biosorbents has been presented, along with their detailed characteristics. The effectiveness of combining the two precursors was confirmed with FTIR, XPS, ^13^C cross polarization/mass angle spinning NMR, and results from elemental analysis. The analysis showed the good electrokinetic properties of the chitin/lignin materials, which will surely be of significant use in industrial applications in which the electrokinetic stability of aqueous dispersive systems plays a crucial role. Moreover, preliminary investigations of the adsorption efficiency of the chitin/lignin sorbents indicates that the materials are suitable for the removal of nickel(II) and cadmium(II) ions from model aqueous solutions. It is noteworthy that the measured values of cadmium(II) adsorption are higher than those for nickel(II), irrespective of the time of adsorption or quantity of sorbent used. The measurements will undoubtedly contribute to the development of research in this area, and will be extended by similar experiments for other metal ions, such as lead, mercury and uranium. In future reports, a significant step will be the determination of kinetic aspects of the removal of selected contaminations from aqueous solution, with consideration given to such parameters as process time, temperature, pH, quantity of biosorbent, and concentration of the model solution.

## References

[1] Gładysz-Płaska A., Majdan M., Pikus S., Sternik D. (2012). Simultaneous adsorption of chromium(VI) and phenol on natural red clay modified by HDTMA. Chem. Eng. J..

[2] Ashrafa M.A., Rehmanb M.A., Aliasa Y., Yusof I. (2013). Removal of Cd(II) onto Raphanussativus peels biomass: Equilibrium, kinetics, and thermodynamics. Desalination Water Treat..

[3] Dhir B., Srivastava S. (2011). Heavy metal removal from a multi-metal solution and wastewater by Salvinianatans. Ecol. Eng..

[4] Jiang M., Jin X., Lu X., Chen Z. (2010). Adsorption of Pb(II), Cd(II), Ni(II) and Cu(II) onto natural kaolinite clay. Desalination.

[5] León-Torres A., Cuerda-Correa E.M., Fernández-González C., Gómez-Serrano V. (2012). On the use of a natural peat for the removal of Cr(VI) from aqueous solutions. J. Colloid Interface Sci..

[6] Zhang A., Lu F., Sun R.C., Ralph J. (2010). Isolation of cellulolytic enzyme lignin from wood dissolved in dimethyl sulfoxide/*N*-methylimidazole. J. Agric. Food Chem..

[7] Monteil-Rivera F., Phuong M., Ye M., Halasz A., Hawari J. (2013). Isolation characterization of herbaceous lignins for applications in biomaterials. Ind. Crop. Prod..

[8] Sarkar S., Adhikari B. (2001). Jute felt composite from lignin modified phenolic resin. Polym. Compos..

[9] Sahoo S., Misra M., Mohanty A.K. (2011). Enhanced properties of lignin-based biodegradable polymer composites using injection moulding process. Compos. Part A.

[10] Carrott P.J., Ribeiro Carrott M.M. (2007). Lignin—From natural adsorbent to activated carbon: A review. Bioresour. Technol..

[11] Guo X., Zhang S., Shan X. (2008). Adsorption of metal ions on lignin. J. Hazard. Mater..

[12] Šćiban M.B., Klašnja M.T., Antor M.G. (2011). Study of the biosorption of different heavy metal ions onto Kraft lignin. Ecol. Eng..

[13] Mohan D., Pittman C.U., Steele P.H. (2006). Single, binary and multi-component adsorption of copper and cadmium from aqueous solutions on Kraft lignin—A biosorbent. J. Colloid Interface Sci..

[14] Shulga G., Shakels V., Skudra S., Bogdanovs V. Modified lignin as an environmentally friendly surfactant. Environment. Technology. Resources. Proceedings of the 8th International Scientific and Practical Conference.

[15] Milczarek G., Inganäs O. (2012). Renewable cathode materials from biopolymer/conjugated polymer interpenetrating networks. Science.

[16] Jesionowski T., Klapiszewski Ł., Milczarek G. (2014). Kraft lignin and silica as precursors of advanced composite materials and electroactive blends. J. Mater. Sci..

[17] Ehrlich H. (2010). Chitin and collagen as universal and alternative templates in biomineralization. Int. Geol. Rev..

[18] Brunner E., Richthammer P., Ehrlich H., Paasch S., Simon P., Ueberlein S., van Pée K.-H. (2009). Chitin-based organic networks: An integral part of cell wall biosilica in the diatom *Thalassiosira pseudonana*. Angew. Chem. Int. Ed..

[19] Goodrich J.D., Winter W.T. (2007). α-Chitin nanocrystals prepared from shrimp shells and their specific surface area measurement. Biomacromolecules.

[20] Sajomsang W., Gonil P. (2010). Preparation and characterization of α-chitin from cicada sloughs. Mater. Sci. Eng. C.

[21] Ehrlich H., Maldonado M., Spindler K.D., Eckert C., Hanke T., Born R., Goebel C., Simon P., Heinemann S., Worch H. (2007). First evidence of chitin as a component of the skeletal fibers of marine sponges. Part I. Verongidae (Demospongia: Porifera). J. Exp. Zool. Part B.

[22] Ehrlich H., Ilan M., Maldonado M., Muricy G., Bavestrello G., Kljajic Z., Carballo J.L., Schiaparelli S., Ereskovsky A., Schupp P. (2010). Three-dimensional chitin-based scaffolds from Verongida sponges (Demospongiae: Porifera). Part I. Isolation and identification of chitin. Int. J. Biol. Macromol..

[23] Wysokowski M., Bazhenov V.V., Tsurkan M.V., Galli R., Stelling A.L., Stöcker H., Kaiser S., Niederschalg E., Gärtner G., Behm T. (2013). Isolation and identification of chitin in three-dimensional skeleton of *Aplysina fistularis* marine sponge. Int. J. Biol. Macromol..

[24] Ehrlich H., Kaluzhnaya O.V., Tsurkan M.V., Ereskovsky A., Tabachnick K.R., Ilan M., Stelling A., Galli R., Petrova O.V., Nekipelov S.V. (2013). First report on chitinous holdfast in sponges (Porifera). Proc. Biol. Sci..

[25] Ehrlich H., Kaluzhnaya O., Brunner E., Tsurkan M.V., Ereskovsky A., Ilan M., Tabachnick K.R., Bazenov V.V., Paasch S., Kammer M. (2013). Identification and first insights into the structure and biosynthesis of chitin from the freshwater sponge *Spongilla lacustris*. J. Struct. Biol..

[26] Yang T.C., Zall R.R. (1984). Absorption of metals by natural polymers generated from seafood processing wastes. Ind. Eng. Chem. Prod. Res. Dev..

[27] Muzzarelli R.A.A. (2009). Chitins and chitosans for the repair of wounded skin, nerve, cartilage and bone. Carbohydr. Polym..

[28] Jayakumar R., Nair A., Sanoj Rejinold N., Maya S., Nair S.V. (2012). Doxorubicin-loaded pH-responsive chitin nanogels for drug delivery to cancer cells. Carbohydr. Polym..

[29] Di Giuseppe A., Crucianelli M., Passacantado M., Nisi S., Saladino R. (2010). Chitin- and chitosan-anchored methyltioxorhenium: An innovative approach for selective heterogenous catalytic epoxidations of olefins. J. Catal..

[30] Filipkowska U. (2008). Desorption of reactive dyes from modified chitin. Environ. Technol..

[31] Tang H., Zhou W., Zhang L. (2012). Adsorption isotherms and kinetics studies of malachite green on chitin hydrogels. J. Hazard. Mater..

[32] Schleuter D., Günter A., Paasch S., Ehrlich H., Kljajić Z., Hanke T., Bernhard G., Brunner E. (2013). Chitin-based renewable materials from marine sponges for uranium adsorption. Carbohydr. Polym..

[33] Li C.B., Hein S., Wang K. (2008). Adsorption of chitin and chitosan. Mater. Sci. Technol..

[34] Lobo-Recio M.A., Lapolli F.R., Belli T.J., Folzke C.T., Tarpani R.R.T. (2013). Study of the removal of residual aluminium though the biopolymers carboxymethylcellulose, chitin and chitosan. Desalination Water Treat..

[35] Kurita K. (2001). Controlled functionalization of the polysaccharide chitin. Prog. Polym. Sci..

[36] Wang X., Xing B. (2007). Importance of structural makeup of biopolymers for organic contaminant sorption. Environ. Sci. Technol..

[37] Klapiszewski Ł., Nowacka M., Milczarek G., Jesionowski T. (2013). Physicochemical and electrokinetic properties of silica/lignin biocomposites. Carbohydr. Polym..

[38] Cárdenas G., Cabrera G., Taboada E., Miranda S.P. (2004). Chitin characterization by SEM, FTIR, XRD, and ^13^C cross polarization/mass angle spinning NMR. J. Appl. Polym. Sci..

[39] Ago M., Jakes J.E., Johansson L.S., Park S., Rojas O.J. (2012). Interfacial properties of lignin-based electrospun nanofibers and films reinforced with cellulose nanocrystals. ACS Appl. Mater. Interfaces.

[40] Wang B., Li J., Zhang J., Li H., Chen P., Gu Q., Wang Z. (2013). Thermo-mechanical properties of the composite made of poly(3-hydroxybutyrate-co-3-hydroxyvalerate) and acetylated chitin nanocrystals. Carbohydr. Polym..

[41] Johansson L.S., Campbell J.M., Koljonen K., Stenius P. (1999). Evaluation of surface lignin on cellulose fibers with XPS. Appl. Surf. Sci..

[42] Briggs D., Grant J.T. (2003). Surface Analysis by Auger and X-ray Photoelectron Spectroscopy.

[43] Wang J., Wang Z., Li J., Wang B., Liu J., Chen P., Miao M., Gu Q. (2012). Chitin nanocrystals grafted with poly(3-hydroxybutyrate-co-3-hydroxyvalerate) and their effects on thermal behavior of PHBV. Carbohydr. Polym..

[44] Oh D., Shin S., Lim C., Hwang D. (2013). Dopamine-mediated sclerotization of regenerated chitin in ionic liquid. Materials.

[45] Haensel T., Comouth A., Lorenz P., Ahmed S.I.U., Krischok S., Zydziak N., Kauffmann A., Schaefer J.A. (2009). Pyrolysis of cellulose and lignin. Appl. Surf. Sci..

[46] Lange P.J., Mahy J.W.G. (1995). ToF-SIMS and XPS investigations of fibers, coatings and biomedical materials. Fresenius J. Anal. Chem..

[47] Stevens J.S., Luca A.C., Pelendritis M., Terenghi G., Downes S., Schroeder S.L.M. (2013). Quantitative analysis of complex amino acids and RGD peptides by X-ray photoelectron spectroscopy (XPS). Surf. Interface Anal..

[48] Spinde K., Kammer M., Freyer K., Ehrlich H., Vournakis J.N., Brunner E. (2011). Biomimetic silicification of fibrous chitin from diatoms. Chem. Mater..

[49] Wen J.L., Sun S.L., Xue B.L., Sun R.C. (2013). Recent advances in characterization of lignin polymer by solution-state nuclear magnetic resonance (NMR) methodology. Materials.

[50] Li J., Revol J.F., Naranjo E., Marchessault R.H. (1996). Effect of electrostatic interaction on phase separation behaviour of chitin crystallite suspensions. Int. J. Biol. Macromol..

[51] Stawski D., Rabiej S., Herczyńska L., Draczyński Z. (2008). Thermogravimetric analysis of chitins of different origin. J. Therm. Anal. Calorim..

[52] Liu Q., Wang S., Zheng Y., Luo Z., Cen K. (2008). Mechanism study of wood lignin pyrolysis by using TG-FTIR analysis. J. Anal. Appl. Pyrolysis.

[53] Shen D.K., Gu S., Luo K.H., Wang S.R., Fang M.X. (2010). The pyrolytic degradation of wood-derived lignin from pulping process. Bioresour. Technol..

[54] Singha B., Kumar Das S. (2013). Adsorptive removal of Cu(II) from aqueous solution and industrial effluent using natural/agricultural wastes. Colloids Surf. B.

